# Structural Similarity Measurement Based Cost Function for Stereo Matching of Automotive Applications

**DOI:** 10.3390/jimaging6080077

**Published:** 2020-08-03

**Authors:** Oussama Zeglazi, Mohammed Rziza, Aouatif Amine, Cédric Demonceaux

**Affiliations:** 1 LRIT, Rabat IT Center, Faculty of Sciences, Mohammed V University, Rabat B.P. 1014, Morocco; rziza@fsr.ac.ma; 2 LGS, National School of Applied Sciences, Ibn Tofail University, Kenitra B.P. 241, Morocco; amine_aouatif@yahoo.fr; 3 ERL VIBOT CNRS 6000, ImViA, Université Bourgogne Franche-Comté, 71200 Le Creusot, France; cedric.demonceaux@u-bourgogne.fr

**Keywords:** stereo matching, structure similarity measurement, cross-based aggregation method, KITTI 2012, KITTI 2015

## Abstract

The human visual perception uses structural information to recognize stereo correspondences in natural scenes. Therefore, structural information is important to build an efficient stereo matching algorithm. In this paper, we demonstrate that incorporating the structural information similarity, extracted either from image intensity (*SSIM*) directly or from image gradients (*GSSIM*), between two patches can accurately describe the patch structures and, thus, provides more reliable initial cost values. We also address one of the major phenomenons faced in stereo matching for real world scenes, radiometric changes. The performance of the proposed cost functions was evaluated within two stages: the first one considers these costs without aggregation process while the second stage uses the fast adaptive aggregation technique. The experiments were conducted on the real road traffic scenes KITTI 2012 and KITTI 2015 benchmarks. The obtained results demonstrate the potential merits of the proposed stereo similarity measurements under radiometric changes.

## 1. Introduction

Intelligent vehicles rely on active sensors (e.g., time-of-flight-camera [1], LiDAR [2]) in order to represent the cloud points of the surrounding environment. However, low cost passive computer vision offers the potential to produce richer geometric representations. In particular, our intention was paid to the stereo matching task, as it is vital for applications that are linked to intelligent vehicles.

The aim of stereo matching process is to estimate the depth of a scene viewed from two stereo images. Stereo matching algorithms can be roughly split into two categories. Sparse algorithms that rely on feature-based matching methods, generally used in camera calibration or orientation tasks [3,4], and dense algorithms, estimate depth values at every pixel value in the image.

Dense algorithms can be classified to global or local approaches. The global approaches formulate the stereo correspondence problem as an energy function over all image pixels with some smoothness constraints. This function is then minimized by global methods, such as the commonly used dynamic programming [5], belief propagation [6], and graph-cuts [7]. Generally, these approaches can effectively alleviate the matching ambiguities and, therefore, provide quite accurate depth results. However, they are inappropriate for real-time applications due to their slow convergence to optimal values. By contrast, local approaches consider for each individual pixel in the image a local smoothness assumption to estimate its depth values [8,9,10]. This makes them computationally inexpensive but produce a lower disparity results, especially in textureless areas. A stereo matching algorithm can be performed in four steps [11]: cost computation, cost aggregation, disparity selection, and disparity refinement. The first step consists of matching pixels of the two stereo pairs. Several cost functions can be adopted in this step. Each of these have different characteristics that enable dealing with specific image regions. The second one, cost aggregation, is performed in order to filter out noisy matches that could have been occurred during the first stage. In the third step, disparity values are selected. The Winner-Take-All (WTA) strategy is often performed. It considers the disparity with the lowest or higher matching cost from the previous aggregation step. The last step, disparity refinement, is optional and it aims to refine erroneous disparity values by filtering out wrong matches using global smoothness assumptions.

Although all of these steps are required for accurate disparity results, the cost computation is the most critical, since early ambiguous cost values considerably affect the accuracy of the final results independently, regardless of the stereo matching algorithm. Therefore, obtaining a robust disparity map in real traffic situations require building a cost function that can be effective under radiometric distortions.

In this paper, we propose two new cost functions, which are based on the structural information (SSIM), $C_{SSIM}$, and its gradient variant, the $C_{GSSIM}$. The performance of the proposed costs was evaluated using both aggregation [10] and no aggregation approaches. The local WTA strategy was adopted to generate disparity maps. The experimental results were conducted on two challenging datasets, the real road traffic stereo pairs of KITTI 2012 [12] and KITTI 2015 [13].

The remainder of the paper is organized, as follows: in Section 2, we review the related works to the matching cost functions. In Section 3, we present the proposed cost function. Experimental results and discussions are given in Section 4. Additionally, finally, we draw conclusions in Section 5.

## 2. Related Work

A wide range of cost functions have been proposed in the literature. Of these, the absolute intensity differences, squared intensity differences, cross correlation sum, and normalized cross-correlation. Non parametric cost functions have been introduced for being robust against radiometric distortions [14]. Authors in [15] have proposed a cost function based on the mutual information in order to handle the complex radiometric relationships between images. Several works have focused on enhancing the performance of the traditional cost functions by proposing enhanced costs or by merging multiple cost functions to provide efficient variants of the existed ones. In [16], the authors fused both the absolute difference on image color and gradient along the horizontal direction. Other studies have exponentially fused the absolute difference on image color with the Census Transform (CT) cost function [17]. The authors in [18] have fused three cost functions: the absolute difference on color image, on image gradients, and the CT computed in image gradients using an exponential function. Authors in [19] have proposed an adaptive fusion method of multiple cost matching functions. The efficiency of the state of art cost function has been widely examined in several studies [20,21,22]. Indeed, the study that is presented in [20] included the comparison of robustness using six cost matching functions in term of photometric distortion and noise. While [21] is more extended and it has included the evaluation of fifteen different cost functions using various optimization schemes. The results have demonstrated that costs that are based on the CT give the best results, particularly for radiometric changes. Recently, authors in [22] have investigated cost functions in stereo matching algorithms for automotive vehicle applications using two different stereo matching algorithms. One is based on global energy optimization (Graph cuts) [7], and the other one uses local adaptive method [10]. The results of this study have proven that the cost function derived from the CT or its variants, as the Cross-Comparison Census (CCC) combined with the mean sum of relative pixel intensity differences within a CT window, provide overly a good performance on the KITTI 2012 benchmark. A variant of CCC cost function [23] was proposed in order to handle better the radiometric distortions. The authors claim that the proposed cost function outperforms the conventional cost functions on the KITTI 2012 benchmark. These studies have demonstrated that it is quite difficult to address the disparity, with radiometric distortions, relying only on intensity-based cost functions. Some research studies have investigated SSIM for stereo matching algorithms. In [24], authors have proposed to compute the final matching cost function using SSIM index over filtered left and right patches obtained from the non-local means algorithm [25]. In [26], the SSIM index has been introduced for multiview setero to compute the matching cost function in coarst-to-fine workflow.

## 3. The Proposed Cost Function

### 3.1. SSIM Based Cost Function ($C_{SSIM}$)

When considering the stereo matching problem as a visual issue. Extracting the most adopted information captured by the Human Visual System (HVS) can provide a consistent information in order to accurately describe the considered patch, and facilitate the matching process. In this context, we propose a new cost function based on the structural information [27]. Let p(x,y) be a pixel in the reference image $\left(I_{1}\right)$, $I_{p}$ is the intensity value of pixel *p* and q(x,y−d) its hypothetical corresponding, with intensity value $I_{q}$ in the target image $\left(I_{2}\right)$ at a disparity *d*. The $C_{SSIM}$ between p and q is defined, as follows:

(1) $$C_{SSIM}\left(p,q,d\right)={\left[l\left(p,q,d\right)\right]}^{\alpha }.{\left[c\left(p,q,d\right)\right]}^{\beta }.{\left[s\left(p,q,d\right)\right]}^{\gamma }$$

where, l(p,q,d) is the luminance, c(p,q,d) the contrast and s(p,q,d) structure measurements between *p* and *q*, defined in Equations (2)–(4), respectively.

(2) $$l\left(p,q,d\right)=\frac{2\mu_{p}\mu_{q}+C}{\mu_{p}^{2}+\mu_{q}^{2}+C}$$

(3) $$c\left(p,q,d\right)=\frac{2\sigma_{p}\sigma_{q}+C}{\sigma_{p}^{2}+\sigma_{q}^{2}+C}$$

(4) $$s\left(p,q,d\right)=\frac{\sigma_{\left(p,q\right)}+C}{\sigma_{p}\sigma_{q}+C}$$

*C* is a small constant to avoid the denominator being zero. $\mu_{p}$ and $\mu_{q}$ are the mean values computed in neighborhood $N_{p}$ and $N_{q}$ of *p* in $I_{1}$ and *q* in $I_{2}$, respectively. $\sigma_{p}$ and $\sigma_{q}$ are standard deviations of *p* and *q* respectively. The standard deviations of *p* in the support window $N_{p}$ is described as follows:

(5) $$\sigma_{p}={\left(\frac{1}{\left(||N_{p}||-1\right)}\sum_{p^{\prime }\in N_{p}}{\left(I_{p^{\prime }}-\mu_{p}\right)}^{2}\right)}^{1/2}$$

where $\left|\right|N_{p}\left|\right|$ is the number of pixels in the support window $N_{p}$. The $\sigma_{\left(p,g\right)}$ is the covariance between *p* and *q*, and can be estimated as:

(6) $$\sigma_{\left(p,q\right)}=\frac{1}{\left(||N_{p}||-1\right)}\sum_{p^{\prime }\in N_{p},q^{\prime }\in N_{q}}\left(I_{p^{\prime }}-\mu_{p}\right)\left(I_{q^{\prime }}-\mu_{q}\right)$$

Finally, α>0, β>0, and γ>0 are parameters that allow for to controlling the influence of the each of the three components.

### 3.2. SSIM Gradient Variant ($C_{GSSIM}$)

Besides the structural information, the human visual system is capable of extracting the image gradients based structural features, such as (edges and points). Thus, in order to take into account this assumption, the structural information is extracted from image derivatives ∂I/∂x, ∂I/∂y, rather than image intensities. To do so, the luminance (l), the contrast (c), and the structure measurement (s), in Equation (1) will be modified by incorporating the gradient. Therefore, the gradient based structural information cost function $C_{GSSIM}$ is defined, as follows:

(7) $$C_{GSSIM}\left(p,q,d\right)={\left[l_{g}\left(p,q,d\right)\right]}^{\alpha }.{\left[c_{g}\left(p,q,d\right)\right]}^{\beta }.{\left[s_{g}\left(p,q,d\right)\right]}^{\gamma }$$

where $l_{g}$, $c_{g}$ and $s_{g}$ are structural information defined as follows:

(8) $$l_{g}\left(p,q,d\right)=\sum_{p\in \left\{\frac{\partial I_{1}}{\partial x},\frac{\partial I_{1}}{\partial y}\right\},q\in \left\{\frac{\partial I_{2}}{\partial x},\frac{\partial I_{2}}{\partial y}\right\}}\frac{2\partial \mu_{p}\partial \mu_{q}+C}{\partial \mu_{p}^{2}+\partial \mu_{q}^{2}+C}$$

(9) $$c_{g}\left(p,q,d\right)=\sum_{p\in \left\{\frac{\partial I_{1}}{\partial x},\frac{\partial I_{1}}{\partial y}\right\},q\in \left\{\frac{\partial I_{2}}{\partial x},\frac{\partial I_{2}}{\partial y}\right\}}\frac{2\partial \sigma_{p}\partial \sigma_{q}+C}{\partial \sigma_{p}^{2}+\partial \sigma_{q}^{2}+C}$$

(10) $$s_{g}\left(p,q,d\right)=\sum_{p\in \left\{\frac{\partial I_{1}}{\partial x},\frac{\partial I_{1}}{\partial y}\right\},q\in \left\{\frac{\partial I_{2}}{\partial x},\frac{\partial I_{2}}{\partial y}\right\}}\frac{\partial \sigma_{\left(p,q\right)}+C}{\partial \sigma_{p}\sigma_{q}+C}$$

$\partial \mu_{p}$ and $\partial \mu_{q}$ are the mean values computed for the neighborhood $\partial N_{p}$ in $\partial I_{1}$ for *p* and $\partial N_{q}$ in $\partial I_{2}$ for *q* and *q* in $\partial I_{2}$. $\partial I_{1}$ and $\partial I_{2}$ are the gradients along x and y directions, respectively. $\partial \sigma_{p}$ and $\partial \sigma_{q}$ are the standard deviations of *p* in $\partial I_{1}$ and *q* in $\partial I_{2}$. The standard deviations of *p* $\partial \sigma_{p}$ is defined, as follows:

(11) $$\partial \sigma_{p}=\sum_{p\in \{\frac{\partial I_{1}}{\partial x},\frac{\partial I_{1}}{\partial y}\}}{\left(\frac{1}{\left(||N_{p}||-1\right)}\sum_{p^{\prime }\in \partial N_{p}}{\left(\partial I_{p^{\prime }}-\partial \mu_{p}\right)}^{2}\right)}^{1/2}$$

The $\partial \sigma_{\left(p,g\right)}$ is defined, as follows:

(12) $$\begin{array}{ccc} \partial \sigma_{\left(p,q\right)} & = & \sum_{p\in \{\frac{\partial I_{1}}{\partial x},\frac{\partial I_{1}}{\partial y}\}}\frac{1}{\left(||N_{p}||-1\right)}\\ & & *\sum_{p^{\prime }\in \partial N_{p},q^{\prime }\in \partial N_{q}}\left(\partial I_{p^{\prime }}-\partial \mu_{p}\right)\left(\partial I_{q^{\prime }}-\partial \mu_{q}\right) \end{array}$$

In contrast to the Equation (1), this enables to compute the new structural features on image principal derivatives with respect to *x* and *y* coordinates.

## 4. Experimental Results

In this section, we evaluate the ability of the proposed cost functions to discriminate stereo correspondences. We explore the proposed costs for stereo matching through two different algorithms: a stereo matching algorithm without aggregation stage and a fast local adaptive aggregation technique. These cost functions are then compared to the top cost functions $C_{DIFFCensus}$[22] and $C_{GCCC}$ [23]. The optimal parameter values that were proposed in [22,23] were retained. Experiments were conducted on the KITTI 2012 [12] and KITTI 2015 [13] training datasets in order to evaluate the proposed approach in the context of intelligent vehicles applications.

- The KITTI 2012 is divided into two sets, training one which contains 194 stereo pairs and 195 stereo pairs in the testing one.
- The KITTI 2015 dataset contains 200 training stereo pairs and 200 testing pairs.

The evaluation for the KITTI 2012 datasets is measured by computing the percentage of disparity errors with respect to the ground truth. While, for the KITTI 2015 *D1*—all error measure is computed, it represents the percentage of pixels for which the estimation error is larger than three pixels and larger than 5% of the ground truth disparity at each pixel. For the parameters sets of both cost functions, $C_{SSIM}$ and $C_{GSSIM}$, were experimentally set as: α=0.9, β=0.1 and γ=0.2 to minimize the overall error rate. Parameter C is set to the smallest value to prevent dividing by zero. In the aggregation stage, the spacial and color similarity thresholds were fixed at *L* = 9 and τ=20, respectively. The local *WTA* strategy was adopted in order to generate disparity results. We used the highest matching cost instead of the lowest one, as the proposed costs are built upon similarity measurement.

### 4.1. Evaluation of the Discriminative Ability of the Proposed Costs

**Figure 1:**
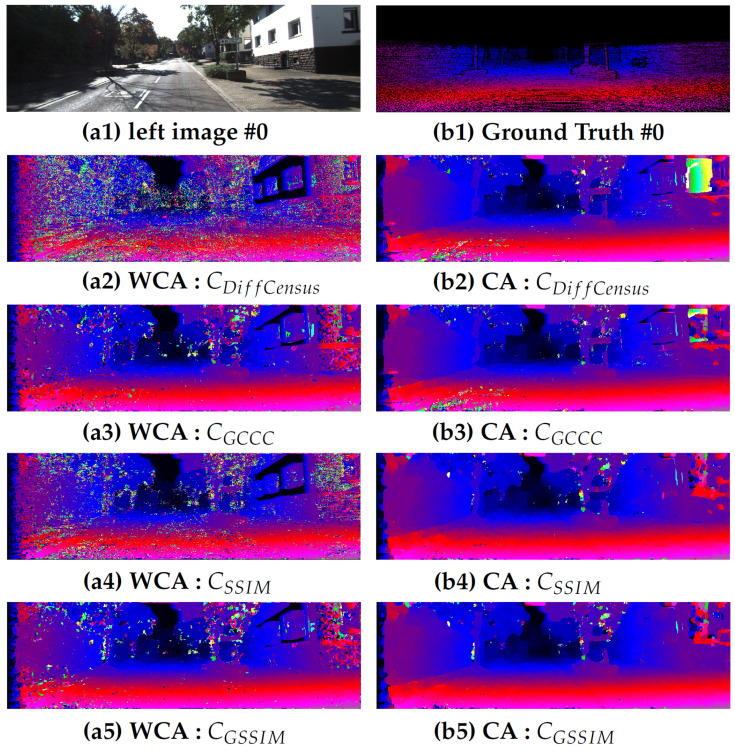
Disparity maps of #0 stereo pair from the KITTI 2012 dataset. The first column corresponds to left stereo (**a1**) with its corresponding ground truth disparity map (**b1**). The computed disparity maps are listed in the following lines corresponding to cost functions $C_{DiffCensus}$, $C_{GCCC}$, $C_{SSIM}$ and $C_{GSSIM}$, respectively. First column (**a**) correspond to the output obtained without the use of an aggregation method (WCA), while second column (**b**) are the output based on the adaptive aggregation method (CA).

In this section, the effectiveness of the proposed cost functions is studied on both KITTI datasets without using any cost aggregation method. Figure 1 shows a visualization of the output disparity results for each cost functions using both two stereo algorithms is presented. Column one shows the results that were obtained without using an aggregation method, while column two shows the results obtained with based on adaptive aggregation method. The output results for the #0 stereo pair from the KITTI 2012 training dataset are presented. The presented figure illustrates, in both cases, that the proposed cost functions lead to promising results, while the conventional costs provide highly noisy disparity results.

Table 1 and Table 2 present the mean error rate on all of the stereo pairs in different regions (non−occluded and all) on both datasets, KITTI 2012 and KITTI 2015, respectively. According to results, our both cost functions achieves notable results. In addition, the $C_{GSSIM}$ provides the lowest rate of error in both datasets.

The presented results demonstrate the discrimination power of the proposed costs without considering aggregation costs, which proves the effectiveness of the SSIM information for capturing reliable local information for stereo matching. The next section investigates the efficiency of these costs while using aggregation techniques.

### 4.2. Evaluation of the Proposed Costs Using the Adaptive Aggregation Technique

To further reduce noise and construct refined cost functions, the adaptive aggregation method [10] was performed. This choice is motivated by the fact that this method is fast and accurate, which is suitable for real time applications.

**Figure 2:**
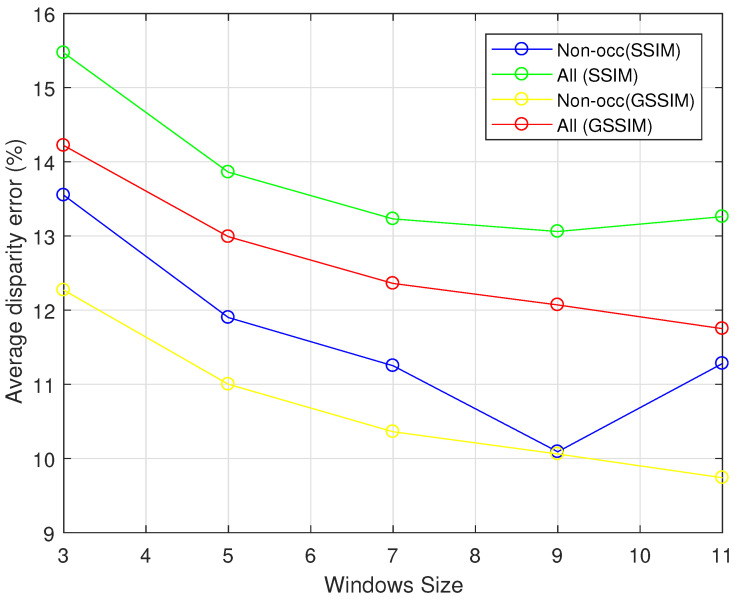
The disparity results obtained with respect to support window size for both cost functions, for KITTI 2012 training sets.

The effectiveness of the proposed method was firstly evaluated with respect to the support window size on KITTI 2012 training datasets. Figure 2 presents the mean error rate, in both non-occluded and all regions, computed at the default 3 pixels threshold for all of training set images. It can be noted that the size of the support window impacts highly the performance of the algorithm of both cost functions. Indeed, significant improvement in the performance of the local stereo matching algorithm can be obtained as the size of the support window increases. More precisely, the improvement is by a factor of 1.65% for the non-occluded and by 1.61% for occluded zones, for the $C_{SSIM}$ cost function when the size window passed from 3 to 5, for example.

In the following, we evaluate the robustness of the proposed cost functions based on adaptive aggregation method against the state-of-the-art cost function. Table 2 and Table 3 present the average percentage of erroneous pixels with both non-occluded and all regions. In Table 3, the errors were calculated at three different pixels error thresholds, while in Table 2 the D1−all error was computed. The obtained results indicate that the proposed $C_{GSSIM}$ cost functions outperform the others ones by a significant margin. Indeed, the $C_{GSSIM}$ provides the lower mean disparity errors on both datasets, followed by the proposed $C_{SSIM}$ cost function under different scenarios. Indeed, in Table 3 at the default three pixel threshold, the improvement obtained by $C_{GSSIM}$ is of the order 2.23, 3.47 for non-occluded region and of 2.84, 3.4 for other zones, with respect to $C_{DIFFCensus}$ and $C_{GCCC}$ costs. Besides, from Table 2, we can see clearly that the performance of our methods are significantly better than all other cost functions in both regions. For example, the improvement obtained by $C_{GSSIM}$ is of the order 1.87, 2.91 for non-occluded region and of 1.82, 2.84 for other zones, with respect to $C_{GCCC}$ and $C_{DIFFCensus}$ costs.

This evaluation shows that the proposed $C_{GSSIM}$ cost function is more appropriate for the real outdoor disparity computation than the top performers $C_{DiffCensus}$ and $C_{GCCC}$.

### 4.3. Sensitivity of the Cost Functions in the Presence of Radiometric Distortions

**Figure 3:**
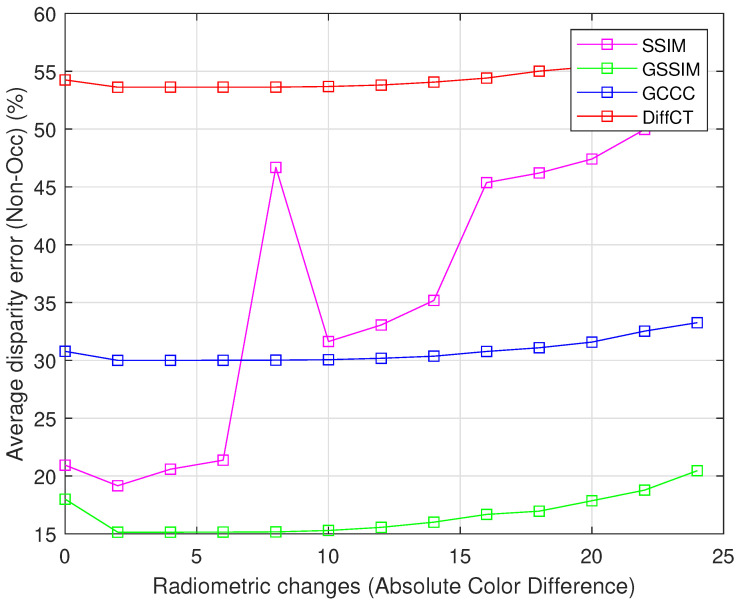
The disparity results obtained with respect to radiometric distortions for all of the presented cost functions, for KITTI 2012 training sets.

In this section, we study the impact of radiometric distortions on different cost functions. These distortions are generated while using the absolute color difference between corresponding pixels [22]. At each level of radiometric distortion, we compute the mean disparity errors for all KITTI training set for $C_{SSIM}$, $C_{GSSIM}$, $C_{DIFFCensus}$[22], and $C_{GCCC}$[23] cost functions. It can be visualized from the Figure 3 that the proposed cost $C_{GSSIM}$ give the lowest error rate at all radiometric distortion levels.

### 4.4. Discussion

In the literature, it has been proven that cost functions based on pixel intensities are very sensitive to radiometric changes. In this paper, new intensity based cost functions have been proposed. It takes the local intensity, luminance, and contrast into account, which provide a significant local information to describe the considered pixel within a support window. This new consideration provides the ability of the proposed cost function to deal with radiometric changes (see Figure 3). The results described in Table 2 and Table 3 demonstrate that the proposed cost functions outperform the top performer, in both KITTI 2012 KITTI 2015 datasets, compared to $C_{DiffCensus}$ and $C_{GCCC}$ costs. Although these latter promise better results with aggregation techniques, the aggregation costs proposed have led to the best results (see Table 2 and Table 3). It must be noted that the overall performance of the proposed cost functions depends on support widow size. It can be seen that both cost functions performs well as the size of the support region increases, as shown in Figure 2. This is trivial since large support regions hold sufficient information to more accurately describe the considered patch, and then lead to good accurate initial cost functions.

## 5. Conclusions

In this paper, we presented a new stereo matching algorithm with a new structural information based cost functions for the cost computation step. Thus, two cost functions were proposed and evaluated using real road scenes from the challenging KITTI 2012 and KITTI 2015 training datasets. The obtained results have demonstrated that both cost functions lead to the lowest disparity mean errors as compared to the top performer in this data set under different scenarios, which has proven that our cost functions are more robust to radiometric distortions than conventional cost functions. The evaluation of the proposed local stereo matching algorithm using the best performing cost function over the current state-of-the-art algorithms has demonstrated the potential merits of the proposed stereo similarity measurement.

## Figures and Tables

**Table 1:** Percentage of erroneous disparities of stereo matching without an aggregation method for KITTI 2012 training database.

Cost Functions	3-px Threshold	
	Non-Occluded	All
$C_{DIFFCensus}$ [22]	54.25	55.30
$C_{GCCC}$ [23]	30.78	32.34
$C_{SSIM}$	20.94	22.73
$C_{GSSIM}$	18.00	19.86

**Table 2:** Results on the KITTI 2015 training datasets.

Cost Functions	No Aggregation Method		Aggregation Method	
	D1—All (Non-Occluded)	D1—All (All)	D1—All (Non-Occluded)	D1—All (All)
$C_{DIFFCensus}$ [22]	50.74	49.87	18.63	20.05
$C_{GCCC}$ [23]	27.52	28.76	14.63	16.05
$C_{SSIM}$	15.23	16.69	11.08	13.06
$C_{GSSIM}$	15.38	16.83	10.06	12.07

**Table 3:** Percentage of erroneous disparities in non-occluded and regions for the KITTI 2012 training set.

Cost functions	2 px Threshold		3 px Threshold		5 px Threshold	
	Non-Occluded	All	Non-Occluded	All	Non-Occluded	All
$C_{DIFFCensus}$ [22]	20.08	21.88	12.97	14.91	9.70	11.63
$C_{GCCC}$ [23]	20.47	22.27	11.93	13.89	10.19	12.18
$C_{SSIM}$	16.39	18.29	11.08	13.06	8.18	10.17
$C_{GSSIM}$	14.06	16.00	10.06	12.07	7.83	9.83
